# *Pediococcus pentosaceus* PR-1 modulates high-fat-died-induced alterations in gut microbiota, inflammation, and lipid metabolism in zebrafish

**DOI:** 10.3389/fnut.2023.1087703

**Published:** 2023-02-01

**Authors:** Yue Liu, Danxu Zhu, Jiwen Liu, Xiaoxia Sun, Feng Gao, Huiping Duan, Lina Dong, Xin Wang, Changxin Wu

**Affiliations:** ^1^Key Lab of Medical Molecular Cell Biology of Shanxi Province, Institutes of Biomedical Sciences, Shanxi University, Taiyuan, China; ^2^The Provincial Key Laboratories for Prevention and Treatment of Major Infectious Diseases Shanxi, Institutes of Biomedical Sciences, Shanxi University, Taiyuan, China; ^3^Department of Internal Medicine, Fourth People's Hospital of Taiyuan, Taiyuan, China; ^4^Central Laboratory, Shanxi Provincial People's Hospital, Taiyuan, China; ^5^State Key Laboratory for Managing Biotic and Chemical Threats to the Quality and Safety of Agro-Products, Institute of Food Research, Zhejiang Academy of Agricultural Sciences, Hangzhou, China

**Keywords:** probiotic, gut microbiota, inflammation, high fat diet, zebrafish

## Abstract

**Introduction:**

Obesity is a health issue worldwide. This study aimed to evaluate the beneficial effects of *Pediococcus pentococcus* PR-1 on the modulating of gut microbiota, inflammation and lipid metabolism in high-fat-diet (HFD)-fed zebrafish.

**Methods:**

Adult zebrafish were fed a commercial (C), high fat (H, 25% fat), probiotic (P, 10^6^ CFU/g), or high fat with probiotic (HP) diets twice daily for 5 weeks. Gut microbiota were analysed using 16S rRNA gene sequencing. Gene expressions of intestinal cytokine, intestinal TJ protein, and liver lipid metabolism were analysed by quantitative real-time polymerase chain reaction. Biochemical and histological analysis were also performed.

**Results and discussion:**

*P. pentosaceus* PR-1 reduced body weight and BMI, indicating its anti-obesity effect. The 16S rRNA sequencing results showed HFD induced a distinct gut microbiota structure from C group, which was restored by probiotic. *P. pentosaceus* PR-1 improved gut health by decreasing the abundance of *Ralstonia* and *Aeromonas* which were increased induced by HFD. Moreover, probiotic restored abundance of Fusobacteria, *Cetobacterium* and *Plesiomonas*, which were decreased in HFD-fed zebrafish. The results of quantitative real-time polymerase chain reaction showed probiotic suppressed HFD-induced inflammation by decreasing the expressions of IL-1b and IL-6. Levels of hepatic TNF-α, IL-1ß, and IL-6 were reduced by probiotic in HFD-fed zebrafish. Probiotic also ameliorated gut barrier function by increasing the expressions of occludin, Claudin-1, and ZO-1. Probiotic exerted anti-adipogenic activity through regulating the expressions of SREBP1, FAS and LEPTIN. Levels of hepatic triglyceride, total cholesterol, low density lipoprotein were also reduced by probiotic. Histological analysis showed probiotic alleviated liver steatosis and injury induced by HFD. *P. pentosaceus* PR-1 might be useful as a dietary health supplement, especially for reducing obesity.

## 1. Introduction

There has been an increasing prevalence of overweight and obesity during the last 50 years, which are characterized as excessive fat accumulation and low-grade systemic inflammatory state (1). Obesity is mainly caused by long-term imbalance between energy intake and energy expenditure, and would increase the risk of a serious chronic diseases including, diabetes, cardiovascular diseases, and cancer (2). Epidemiological and animal studies have shown that high-fat diet (HFD) is associated with body weight gain and would promote obesity (3, 4).

Several studies have identified the alterations in the gut microbiota, immune response and metabolic functions induced by HFD and obesity (4–6). The increased ratio of Firmicutes/Bacteroidetes is one of primary changes induced by a consumption of HFD in human and animal studies (7). Velázquez et al. (8) found long-term HFD in mice could lead to pathophysiological and microbial changes that observed in non-alcoholic fatty liver disease patients. The increased relative abundance of Firmicutes phylum, *Adercreutzia, Coprococcus, Dorea*, and *Ruminococcus* species, and decreased relative abundance of *Turicibacter* and *Anaeroplasma* were observed in HFD mice. Moreover, a higher ratio of Firmicutes/Bacteroidetes was found in HFD mice (8). Microbiota changes contributing to the increase of Firmicutes/Bacteroidetes ratio may arise from the increases in Erysipelotrichales, Bacilli, and Clostridiales (belonging to Firmicutes phylum) (9). The increased abundance of *Dorea* and *Ruminococcus* (belonging to Firmicutes phylum) also contribute to the increased ratio of Firmicutes/Bacteroidetes (8–10). A consumption of HFD could also lead to the increased abundance of Proteobacteria, which could promote intestinal inflammation and induce a mucosal proinflammatory immune response (11). The decrease in Prevotellaceae and Rikenellaceae abundance is also observed in HFD-fed rats (12, 13). In addition, HFD often drive a decrease in abundance of *Bifidobacterium* spp., which is considered to be beneficial to host health and negatively correlated with metabolic endotoxemia (14). Besides, HFD could also directly or indirectly perturb the gut barrier function and increase the intestinal permeability through suppressing the expression of intestinal tight junction (TJ) proteins, such as occludin, claudin, and zona occludens (ZO) (15–17). A consumption of HFD could also disrupt immune homeostasis and promote intestinal inflammation, with increased levels of proinflammatory cytokines tumor necrosis factor α (TNF-α), interleukins (IL)-1β, and IL-6 (2, 18, 19).

Probiotics are live microorganisms, conferring beneficial effects to host health when administered in adequate amounts (20, 21). Several studies have shown that probiotics could positively modulate the gut microbial composition, immune, and lipid metabolism markers and following a HFD (22–26). *Pediococcus pentosaceus*, one type of LAB, has attracted much attention in recent years. *P. pentosaceus* LI05, isolated from a heathy volunteer fecal sample, could protect against *Clostridium difficile* infection (27–29). In addition, *P. pentosaceus* has been shown to reduce oxidative stress (30), alleviate constipation (31), liver (32, 33) and reproductive system injury (34), and improve hyperlipidaemia (35). *P. pentosaceus* PP04 isolated from the Northeast pickled cabbage could ameliorate hyperlipidemia by the AMPK signaling pathway and improve oxidative stress in HFD mice (35). In addition, PP04 could improve gut barrier integrity by stimulating the expressions of TJ proteins, subsequently decreasing levels of hepatic lipopolysaccharides, alanine aminotransferase, and aspartate aminotransferase in HFD mice (35). Moreover, PP04 could relive intestinal inflammation by the NF-κB/Nrf2 signaling pathway, and restore the gut microbiota homeostasis in HFD mice (36). A clinical trial found that heat-killed *P. pentosaceus* LP28 showed an antiobesity effect and reduced BMI, body fat and waist circumference. *P. pentococcus* PR-1 is a probiotic strain that was isolated from an adult fecal sample in our laboratory. It was found to be acid and bile resistant, and its metabolites showed antioxidant, and antibacterial properties (37). However, the functional effect of *P. pentosaceus* PR-1 has not previously been investigated as probiotics in *in vivo* studies of HFD-induced obesity.

Several studies have found zebrafish serve as a useful animal model of human HFD-induced obesity for research into the alterations of gut microbiota community and inflammation driven by HFD due to its similarity of organs to humans, the availability of a complete genome sequence, and its highly conserved physiological pathways (2, 38–43). Although zebrafish is dominated by Proteobacteria, mice and human are dominated by Firmicutes and Bacteroidetes, the response to microbiota colonization are similar (44).

The current study aimed to investigate the influence of *P. pentosaceus* PR-1 isolated from human feces on gut microbiota composition, intestinal permeability and inflammation, and lipid metabolism in HFD-fed zebrafish.

## 2. Materials and methods

### 2.1. Chemicals

Bacterial media were obtained from Oxoid Ltd. (Basingstoke, UK). Unless stated differently, chemicals were obtained from Sangon Biotech (Shanghai, China). Polymerase Chain Reaction (PCR) primers were synthetized from Sangon Biotech (Shanghai, China).

### 2.2. Probiotic strain

*P. pentosaceus* PR-1 (Genebank ID: MW800163) was a potential probiotic isolated from healthy adult feces and stored in our laboratory. It was cultured in de Man Rogosa and Sharpe (MRS) broth/agar at 37°C. The cells were harvested from 1 L of 24 h culture of *P. pentosaceus* PR-1 by centrifuge at 5,000 × g, 20 min, 4°C. Next, the cells were washed twice with sterile PBS and resuspended in skim milk medium contained 10% skim milk and 5% sucrose (cryoprotectant). The suspension was frozen at −20°C for 4 h and at −80°C overnight, and then transferred into a Modulyo bench top freeze dryer (Edwards, UK; −45°C, 48 h, 10^−1^ torr) for lyophilization. Upon completion, the lyophilized probiotic strain was retrieved and stored at 4°C for further use.

### 2.3. Zebrafish care and diets

Three-month-old adult zebrafish (*Danio rerio*) were kept in a zebrafish breeding recirculating system under a 14-h light−10-h dark cycle at 28°C. The zebrafish were fed twice daily at 09:00 a.m. and 17:00 p.m. with a commercial diet (Tetra Bits Complete) according to standard protocol (45). One week before the experiment, the male zebrafish were selected for the treatments to avoid the sex differences in metabolism. Four experimental groups (30 zebrafish per group) were evaluated: control (C), high fat (H), probiotic (P), and HFD supplemented with probiotic (HP). Zebrafish of each group were kept in a 4 L tank. C group were fed with a commercial diet (Tetra Bits Complete), and the commercial diet was prepared in Milli-Q water (1 g in 100 mL water) and autoclaved. H group were fed with HFD, which consisted of a commercial diet supplemented with 25% (w/w) of lard. The lard was melted and added to the liquid control diet previously to the sterilization. P group were fed with a commercial diet supplemented with probiotic. Lyophilized probiotic *P. pentosaceus* PR-1 was added to the sterilized liquid control diet at a final concentration of 10^6^ CFU/g. For HP group, *P. pentosaceus* PR-1 (10^6^ CFU/g) were added to the HFD. During experiment, zebrafish were fed with the diets at a ratio of 4% of their average body weight twice daily at 09:00 a.m. and 17:00 p.m. during the feeding period, and water was changed daily at the end of the day. Five zebrafish were collected randomly form each group and weighted every week. Meanwhile, the body length (mm) was also recorded and the body mass index (BMI, body weight/body length^2^) was calculated as described (38). After 5 weeks, the zebrafish were anesthetized using an ice bath and sacrificed for specimens. This study was performed in strict accordance with the Laboratory Animal Guideline for Ethical Review of Animal Welfare (GB/T 35892-2018, China) and was approved by the Experimental Animal Welfare and Ethics Committee of the Shanxi University (SXULL2019004).

### 2.4. Gut microbiota analysis

Gut microbiota were analyzed using 16S rRNA gene sequencing. Intestinal content samples were collected at 4–6 h post the last feeding. The intestinal contents of three zebrafish were pooled as one sample and total intestinal bacterial DNA was extracted by using TIANamp Bacteria DNA Kit (TianGen, Beijing, China) according to the instruction of manufacturer. Universal primers 343F (5′-TACGGRAGGCAGCAG-3′) and 798R (5′-AGGGTATCTAATCCT-3′) were used to amplify the V3–V4 regions of 16S rRNA genes. The high-throughput 16S rRNA gene sequencing of the samples was performed by commercial company Personalbio Co., Ltd. (Shanghai, China). Sequencing was conducted on a MiSeq PE300 platform (Illumina) using the 2 × 250 bp paired-end protocol by Personalbio. A sample barcode was contained in the reverse primer, and both primers were linked with Illumina sequencing adapters. After sequencing the raw data were saved in the FASTQ format. With application of sliding window trimming method and Trimmomatic software (version 0.35), the sequence data were scanned to discard sequences with quality score below 20 and shorter than 50 bp. Afterwards, Flash software (version 1.2.11) and split_libraries software (version 1.8.0) in QIIME was used to assemble the paired-end reads and obtain clean tags. The chimeras in clean tags were removed by UCHIME (version 2.4.2) software and valid tags were obtained. Then Vsearch (version 2.4.2) software was used to form operational taxonomic units (OTUs) with 97% similarity cutoff, and sequence with the highest abundance was selected as the representative of this OTU. RDP classifier Naive Bayesian was used to blast representative reads against Silva database (version 123), and annotation of OTUs was confirmed.

### 2.5. Bioinformatics analysis

Analysis of sequencing data analyses were mainly performed using QIIME2 2019.4 and R packages (v.3.2.0). The alpha diversity and beta diversity based on Bray–Curtis metrics was analyzed with QIIME2 following the standard protocol (46). Taxa abundances at the at the genus levels were statistically compared among groups by MetagenomeSeq and visualized as Manhattan plots. Linear discriminant analysis (LDA) coupled with effect size measurements (LEfSe) were performed to obtain the important indicator taxa with significant changes in relative abundance among groups. Microbial functions were predicted using PICRUSt2 (47) against KEGG (https://www.kegg.jp/), and Metacyc (https://metacyc.org/) databases using default parameters.

### 2.6. Quantitative real-time polymerase chain reaction analysis

After 24 h fast, six fish from each tank were randomly sampled for gene expression analysis of intestinal cytokine, intestinal TJ protein, and liver lipid metabolism. Total RNA from intestine and liver were extracted using TRIzol method. One microgram RNA from each sample was transformed into cDNA. SYBR Green SuperReal PreMix Plus (TianGen, Beijing, China) was used for qRT-PCR with cDNA as template. ß-actin was used as the reference gene to normalize the mRNA levels of the target gene, and then the fold changes were calculated by 2^−ΔΔCt^ method. The detailed methods were described as previously (48). The primer sequences were listed in Table 1.

**Table 1:** The primer sequences for quantitative (qPCR) analysis of intestinal inflammation, tight junction proteins, and lipid metabolism-related genes.

**Gene**	**Forward primer (5^′^-3^′^)**	**Reverse primer (5^′^-3^′^)**
TNF-α	AGGCAATTTCACTTCCAAGG	AGGTCTTTGATTCAGAGTTGTATCC
IL-1β	ATCCAAACGGATACGACCAG	TCGGTGTCTTTCCTGTCCAT
IL-6	TCAACTTCTCCAGCGTGATG	TCTTTCCCTCTTTTCCTCCTG
Occludin	AGATGTGGAGGACTGGGTCA	ATTACGGACGGGCAGAATC
Claudin-1	CTGCTGTATCTGTGGGAGTGAA	TAATCAGGAGAACAGGCGAAG
ZO-1	ACAAGAACAGGGCGGAACAGT	ACCTCCAGAAATCAGCACGA
SREBF1	CATCCACATGGCTCTGAGTG	CTCATCCACAAAGAAGCGGT
FAS	GAGAAAGCTTGCCAAACAGG	GAGGGTCTTGCAGGAGACAG
LEPTIN	AGCTCTCCGCTCAACCTGTA	CAGCGGGAATCTCTGGATAA
β-Actin	ATGAAGATCCTGACCGAG	TAGCTCTTCTCCAGGGAG

### 2.7. Biochemical analysis

The liver tissue of three zebrafish were pooled as one sample, homogenized in ice-cold PBS, and centrifuged at 13,000 × g, 10 min, 4°C, giving totally three mixed samples for biochemical analysis. Supernatants were used to determine concentrations of triglyceride, total cholesterol, low density lipoprotein (LDL) using commercial kits (Nanjing Jiancheng Technology, China), and TNF-α, IL-1ß, IL-6 using ELISA kits (Shanghai Qingqi Biotechnology, China), following the manufacturer's instructions. The samples were tested in triplicate, and the average values was used as the result.

### 2.8. Histological analysis

The liver tissues were collected from three fish per treatment. The samples were rinsed with sterilized PBS and fixed in 4% paraformaldehyde in PBS, then embedded in paraffin, sectioned, and stained with hematoxylin and eosin (H&E). Images were obtained using microscope (Leica DMIL-LED, Germany).

### 2.9. Statistical analysis

Statistical analysis was carried out by using SPSS 21.0 statistical software. The results are expressed as mean ± standard deviation (SD) and were analyzed by one-way ANOVA. Significant differences were assessed by *post-hoc* Tukey HSD (Honestly Significant Difference) test. A value of *p* < 0.05 indicates there was a significant difference.

## 3. Results

### 3.1. *P. pentosaceus* PR-1 suppresses HFD-induced obesity in zebrafish

**Figure 1:**
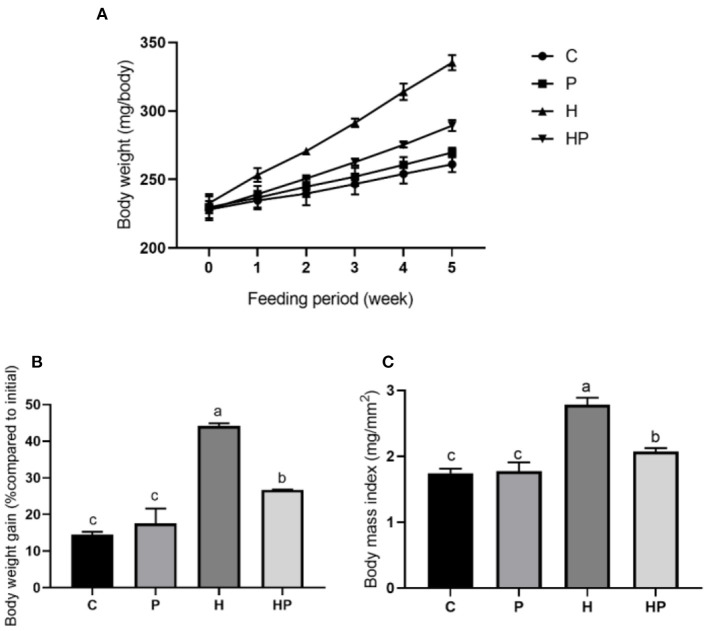
Changes in body weight and BMI in zebrafish on the different diets. **(A, B)** Changes in body weight during 5 weeks. **(C)** BMI in adult zebrafish after 5 weeks.

This study aimed to explore the beneficial effects of *P. pentosaceus* PR-1 in HFD-fed zebrafish, and alterations in body weight and BMI were measured. As shown in Figure 1, zebrafish in all groups have gained weight significantly compared to their baseline weights after 5 weeks. The H group had a significantly higher body weight gain (22.14%) than the C group (*p* < 0.05; Figures 1A, B). *P. pentosaceus* PR-1 could attenuate zebrafish body weight gain; the body weight of HP group was 12.22% higher than that of C group after 5 week feeding (Figures 1A, B). Similarly, at the end of the experiment, H group showed the highest BMI (2.780 mg/mm^2^) compared to the other three groups, and *P. pentosaceus* PR-1 could attenuate zebrafish BMI (Figure 1C). The results indicated that the obesity model in zebrafish was successfully established following 5 week feeding of HFD. Moreover, the results indicated that the probiotic strain *P. pentosaceus* PR-1 has an anti-obesity activity due to their suppressing effects on body weight gain and BMI in HFD-fed zebrafish.

### 3.2. P. *pentosaceus* PR-1 modulates the gut microbiota of HFD-fed zebrafish

**Figure 2:**
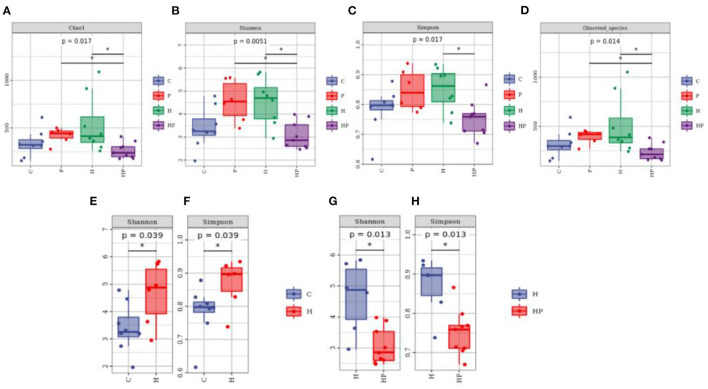
Boxplots of alpha diversity indices of gut microbiota community based on 16S rRNA sequencing. **(A–D)** Gut microbial alpha diversity of C, P, H and HP groups based on **(A)** Chao1, **(B)** Shannon, **(C)** Simpson, and **(D)** Observed species. All data are normalized to C group (100%) (*p < 0.05). **(E, F)** Gut microbial alpha diversity of C and H groups based on **(E)** Shannon, and **(F)** Simpson. All data are normalized to C group (100%) (*p < 0.05). **(G, H)** Gut microbial alpha diversity of H and HP groups based on **(G)** Shannon and **(H)** Simpson. All data are normalized to H group (100%) (*p < 0.05).

**Figure 3:**
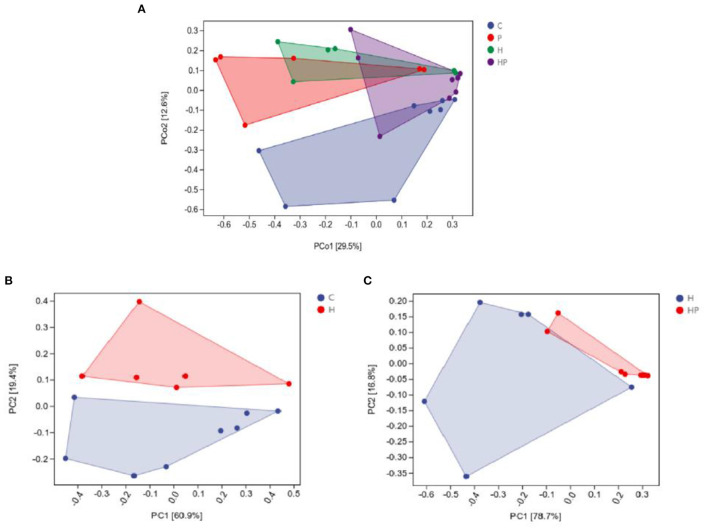
Principal coordinate analysis (Bray-Curtis dissimilarity) score plot of gut microbiota of the zebrafish fed on four different groups **(A)**, on C and H group **(B)**, and on H and HP group **(C)**.

The alpha diversity of different groups was assessed using Chao 1, Shannon index, Simpson index, and observed richness (Figures 2A–H). Probiotic administration increased alpha diversity with higher levels of four indexes, but not significant compared to C group. In addition, the alpha diversity analysis revealed that the microbiota of H group had significantly higher levels of Shannon and Simpson indexes compared to C group (Figures 2E, F), and *P. pentosaceus* PR-1 supplementation induced significantly decrease in Shannon and Simpson indexes in zebrafish fed with HFD (Figures 2G, H). We then analyzed beta diversity by Principal coordinates analysis (PCoA) (Bray-Curtis dissimilarity distance) (Figures 3A–C). Samples representing zebrafish fed with a normal diet and HFD showed distinct clustering pattern on the PCoA score plot (Figures 3A, B), suggesting differences in the gut microbiota structure between C and H groups. Anosim analysis showed that there was difference between the C and H groups (Bray-Curtis: *P* = 0.065; Supplementary Table 1). In addition, probiotic supplementation would restore the microbiota structure in HFD-fed zebrafish to some extent (Figure 3A), suggesting the ability of probiotic to restore the gut microbiota diversity, which was disrupted by HFD. Anosim analysis showed that there was significant difference between the H and HP groups (Bray-Curtis: *P* = 0.016; Supplementary Table 2).

**Figure 4:**
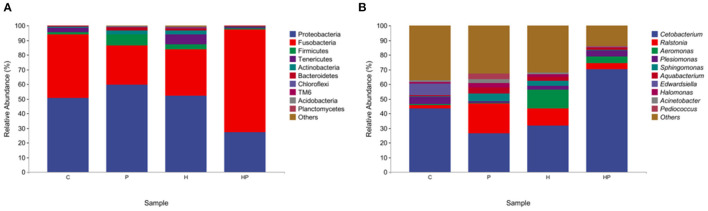
The gut microbiota of zebrafish in C, P, H, and HP groups. Relative abundance at phylum **(A)** and genus **(B)** level of the gut microbiota.

At the phylum level, Proteobacteria and Fusobacteria were dominated in the intestine, with a total proportion of over 80%. *P. pentosaceus* PR-1 supplementation group showed higher relative abundance of Fusobacteria than that of H group. The relative abundance of Proteobacteria and Tenericutes was decreased in probiotic supplementation group compared to H group (Figure 4A). At genus level, HFD induced an increased in the relative abundance of *Ralstonia, Aeromonas, Sphingomonas* and a decrease in the relative abundance of *Cetobacterium, Plesiomonas*. The zebrafish fed with probiotic had the highest relative abundance of *Pediococcus*. More importantly, the probiotic supplementation in HFD-fed zebrafish increased and restored the relative abundance of *Cetobacterium* and *Plesiomonas*. The proportions of *Ralstonia, Aeromonas, Sphingomonas* were decreased in HP group compared to H group (Figure 4B). MetagenoeSeq analysis was used to identify the phyla and genera that were significantly upregulated between the C group and H group, and between H group and HP group. Compared to C group, HFD induced significant upregulation of *Pseudonocardia, Staphylococcus*, and *Aeromonas* in H group (Supplementary Figure 1A). Compared to H group, probiotic supplementation significantly upregulated the abundance of *Pediococcus* and *Cetobacterium* (Supplementary Figure 1B).

**Figure 5:**
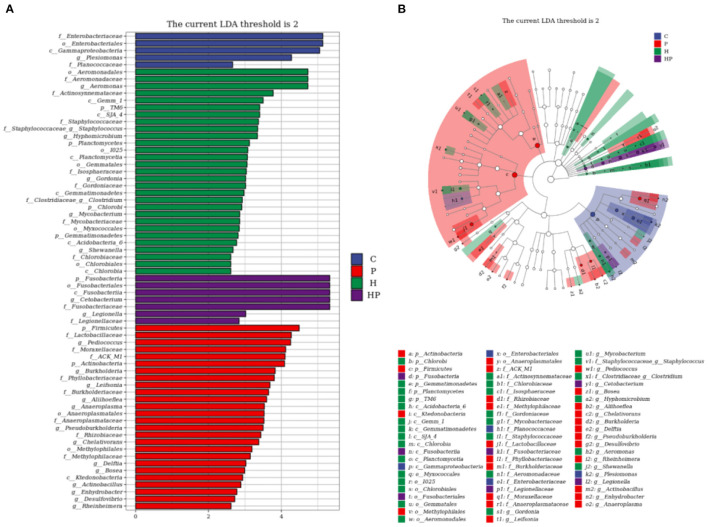
LEfSe analysis explored the discriminative microbiota in group C, P, H, and HP, respectively. **(A)** The LDA score histogram; **(B)** the cladogram. *p* < 0.05 was used as a threshold for LEfSe analysis.

LEfSe analysis was used to explore the discriminative biomarker taxa in each group (Figure 5). In LDA score histogram, different stable taxa in different groups are marked with different colors, and the higher the LDA score value, the deeper the impact degree. According to Figure 5A, there were 5, 29, 7, and 26 different bacteria taxa in group C, H, HP and P, respectively. Compared to C group, *Pseudonocardia, Staphylococcus, Aeromonas*, and *Streptococcus* were enriched in H group, represented as the biomarker species induced by HFD (Supplementary Figure 2A). *Cetobacterium* and *Pediococcus* were enriched in HP and P groups, respectively. Compared to H group, *Cetobacterium* and *Pediococcus* were enriched in HP group, represented as the biomarker species induced by probiotic (Supplementary Figure 2B). In cladogram, the points scattering from the inside out represent the level of taxa from kingdom to genus (Figure 5B). The blue, red, green, and purple points represent the biomarker microbiota in group C, P, H, and HP, respectively. The biomarker genus of group HP mainly belonged to Fusobacteria phylum, and those of group H belonged to Gammaproteobacteria and Staphylococcaceae (Figure 5B).

**Figure 6:**
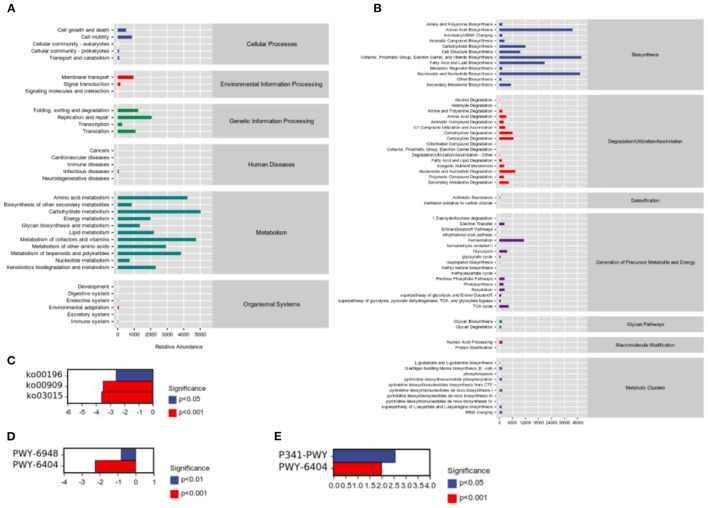
Sample functional abundances predicted by PICRUSt2 based on the abundance of labeled gene sequences in the samples. **(A)** KEGG databases. **(B)** Metacyc databases. **(C)** Significant pathway analysis between H and HP groups based on KEGG databases. **(D)** Significant pathway analysis between C and H groups based on Metacyc databases. **(E)** Significant pathway analysis between H and HP groups based on Metacyc databases.

While studying microbial ecology, the functional potential of microbiota was also investigated. PICRUSt2 software predicts sample functional abundances based on the abundance of labeled gene sequences in the samples. All samples were predicted through the KEGG, and Metacyc databases. We observed the most enrichment in the metabolism of carbohydrates, amino acids, cofactors, and vitamins (Figure 6A). In addition, we found that cofactors, the prosthetic group, electron carriers, vitamin biosynthesis, nucleoside and nucleotide biosynthesis, and amino acid biosynthesis were abundant (Figure 6B). Besides, there were significant differences in pathways were detected between groups. According to results of predicted from KEGG databases, compared to H group, HP significantly downregulated photosynthesis—antenna proteins (ko00196, *P* = 0.000472), sesquiterpenoid biosynthesis (ko00909, *P* = 3.23 × 10^−8^), and mRNA surveillance pathway (ko03015, *P* = 6.67 × 10^−6^; Figure 6C). According to results of predicted from Metacyc databases, compared to C group, H significantly upregulated glycolysis V (Pyrococcus) (P341-PWY, *P* = 9.25 × 10^−5^), and superpathway of mycolyl-arabinogalactan-peptidoglycan complex biosynthesis (PWY-6404, *P* = 4.45 × 10^−9^; Figure 6D). Compared to H group, HP significantly downregulated sitosterol degradation to androstenedione (PWY-6948, *P* = 1.85 × 10^−8^), and superpathway of mycolyl-arabinogalactan-peptidoglycan complex biosynthesis (PWY-6404, *P* = 1.62 × 10^−14^; Figure 6E).

### 3.3. P. *pentosaceus* PR-1 regulated intestinal inflammation, gut permeability, and lipid metabolism in HFD-fed zebrafish

**Figure 7:**
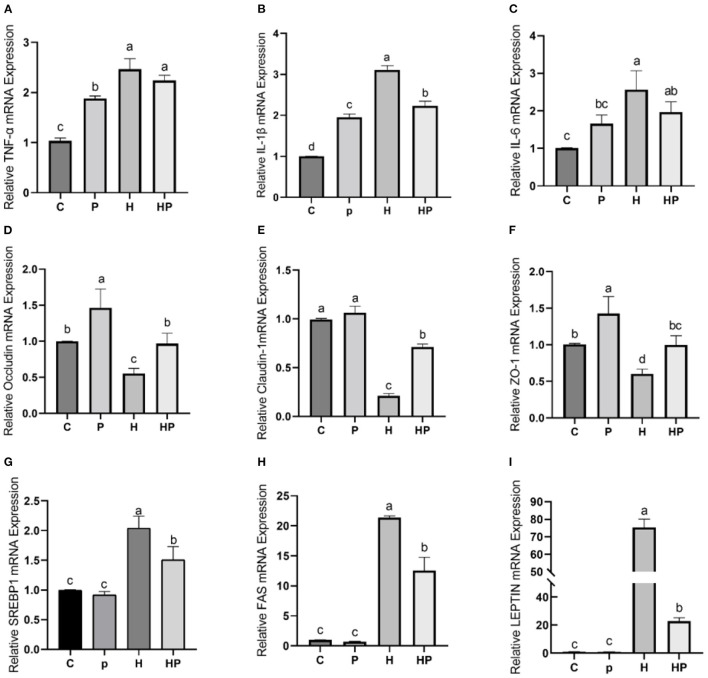
Effect of *P. pentosaceus* PR-1 on intestinal inflammation, gut permeability, and lipid metabolism in HFD-fed zebrafish. **(A–C)** The total RNA was extracted from the intestine, and the relative mRNA expression levels of TNF-α **(A)**, IL-1ß **(B)**, and IL-6 **(C)** were determined by qRT-PCR. **(D–F)** The total RNA was extracted from the intestine, and the relative mRNA expression levels of Occludin **(D)**, Claudin-1 **(E)**, and ZO-1 **(F)** were determined by qRT-PCR. **(G, H)** The total RNA was extracted from the liver, and the relative mRNA expression levels of SREBP1 **(G)**, FAS **(H)**, and LEPTIN **(I)** were determined by qRT-PCR. Values were represented as the mean ± S.D. (*n* = 6). Values with different superscript letters are significantly different (*p* < 0.05).

**Figure 8:**
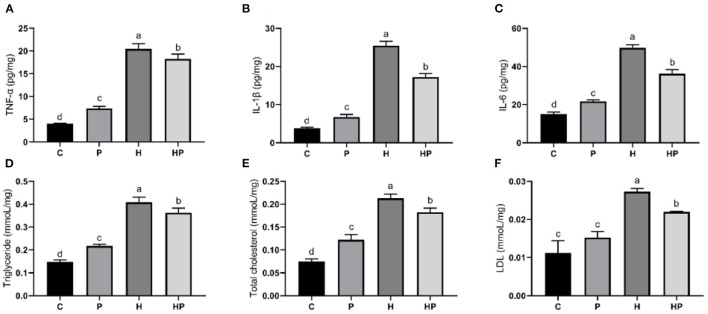
Effect of *P. pentosaceus* PR-1 on liver pro-inflammatory cytokines **(A–C)** and biochemical parameters **(D–F)** in HFD-fed zebrafish. **(A)** Hepatic concentration of tumor necrosis factor α (TNF-α). **(B)** Hepatic concentration of interleukin-1ß (IL-1ß). **(C)** Hepatic concentration of interleukin-6 (IL-6). **(D)** Hepatic level of triglyceride. **(E)** Hepatic level of total cholesterol. **(F)** Hepatic level of low-density lipoprotein (LDL). Values were represented as the mean ± S.D. (*n* = 3). Values with different superscript letters are significantly different (*p* < 0.05).

Considering the influence of inflammation on the level of adipokine, the relative expressions of inflammatory genes encoding TNF-α and interleukins (IL-1β, IL-6) in all treatment groups were evaluated. As shown in Figures 7A–C, HFD increased all tested genes encoding pro-inflammatory cytokines compared to the C group. Probiotic supplementation could significantly down-regulate the expression of IL-1β and IL-6 (Figures 7B, C). In addition, the hepatic levels of pro-inflammatory cytokines were shown in Figures 8A–C. Compared to the C group, the concentrations of TNF-α, IL-1ß, and IL-6 were significantly elevated in zebrafish fed with HFD. The probiotic supplementation decreased the hepatic concentrations of TNF-α, IL-1ß, and IL-6 in HF group. The results indicate that *P. pentosaceus* PR-1 attenuated intestinal inflammation induced by HFD.

The results of qRT-PCR also showed that HFD dramatically decreased relative expression of all tested genes encoding TJ proteins, including Occludin, Claudin-1, and Zonula occludens-1 (ZO-1) compared to the C group (Figures 7D–F). Notably, *P. pentosaceus* PR-1 supplementation could restore their expressions in HFD-fed zebrafish (Figures 7D–F).

Furthermore, the regulatory effects of *P. pentosaceus* PR-1 on lipid metabolism were also investigated. Elevated levels of triglyceride, total cholesterol and LDL were observed in zebrafish fed with HFD (Figures 8D–F). Probiotic supplementation significantly reduced the hepatic triglyceride, total cholesterol, and LDL compared with those in the HF group, whereas did not return their levels to normal compared to C group (Figures 8D–F). In addition, the relative expressions of genes encoding lipid metabolism markers, such as sterol regulatory element binding proteins (SREBP1), fatty acid synthase (FAS) and leptin (LEPTIN) in all treatment groups were evaluated by qRT-PCR. As shown in Figures 7G–I, the relative expressions of SREBP1, FAS, and LEPTIN were significantly higher in HFD-fed zebrafish compared to zebrafish in group C, P, and HP. Moreover, HFD mostly strongly stimulated the expressions of LEPTIN. Probiotic supplementation significantly inhibited the expressions of SREBP1, FAS, and LEPTIN which were induced by HFD, suggesting the anti-adipogenic activity of *P. pentosaceus* PR-1.

### 3.4. Effect of *P. pentosaceus* PR-1 on liver injury in HFD-fed zebrafish

**Figure 9:**
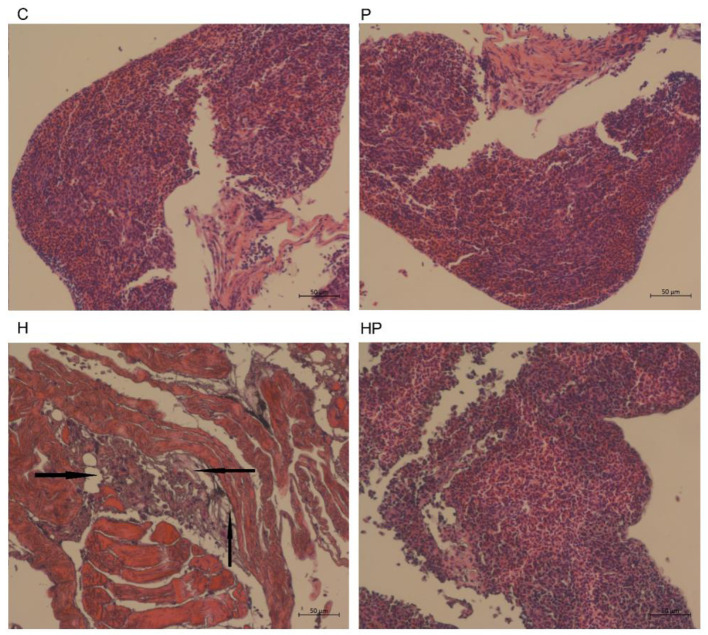
Histological changes of liver sections measured by H&E staining (scale bar is 50 μm). The black arrows indicate extensive hydropic degeneration of hepatocytes characterized by swollen, pale, vacuolated cytoplasm.

It could be observed from the H&E staining of the liver sections (Figure 9) that the boundary was blurred and the integrity of the hepatic cell was damaged in H group, indicating the liver injury in HFD-fed zebrafish. More importantly, HFD detrimentally contributed to substantial accumulation of fat in the liver in H group, whereas probiotic could effectively attenuate the fat accumulation caused by HFD (Figure 9), indicating that *P. pentosaceus* PR-1 supplementation ameliorated liver injury caused by HFD.

## 4. Discussion

Previous studies showed that probiotics could prevent and ameliorate obesity through regulating microbiota composition, suppressing metabolic inflammation and improving lipid metabolism (22–26). Recently, *P.pentosaceus*, one type of LAB, has drawn much attention due to its antimicrobial and antioxidant activities, immunoregulatory activity and therapeutic effects on colitis, constipation, liver injury, and hyperlipidaemia (49–52). However, only a few works have investigated its anti-obesity effect. Zebrafish have similar morphology, physiology, and functions with mammals and have been considered as a useful *in vivo* model for HFD-induced obesity research (2, 38–43). Although the temperature of zebrafish intestine is not optimum for the growth of probiotics such as *Lactobacillus* sp., *Bacillus* sp., *Bifidobacterium* sp., and *Pediococcus*, a rising number of studies have applied zebrafish as model to investigate their potentials. *Bacillus coagulans* 09·712 and *Lactobacillus plantarum* 08·923 showed robust zebrafish gut adhesion capability, and played immunoregulatory and immunoprotective roles in effective stimulation of anti-inflammatory response and barrier regeneration within the mucosa to protect zebrafish against infection (53). *Bifidobacterium lactis* BL-99 also could promote the intestinal integrity, improved the histopathology of the adult zebrafish intestinal inflammation, increased the goblet cell numbers, and recover microbiota metabolism to maintain intestinal health (54). Besides, several studies have found *Pediococcus* could exert beneficial effects in zebrafish (55–58). A previous study found *P. pentosaceus* YC enhanced host resistance against *Aeromonas hydrophila* infection through increasing intestinal butyrate, IL-1β production, and raising intestinal neutrophil level (50). Therefore, *P. pentosaceus* could exert beneficial effect in zebrafish, subsequently the current study investigated the beneficial effects of *P.pentosaceus* PR-1 in a HFD-induced obesity zebrafish model.

Here, we found that probiotic supplementation could ameliorate HFD-induced obesity, reflected by the decreased body weight gain and BMI in HP group compared with H group. This was in agreement with a clinical trial, that *P. pentosaceus* LP28 displayed an anti-obesity effect through decreasing BMI, body fat and waist circumference of subjects (59).

Previous studies showed that HFD could detrimentally influence gut health by disrupting gut microbiota homeostasis (2, 41). In the present study, HFD induced a distinct gut microbiota structure from C group, which was restored by *P. pentosaceus* PR-1 supplementation. Proteobacteria and Fusobacteria have been reported to be the predominant phyla in zebrafish intestine (48). Compared with the H group, there was an increased abundance of Fusobacteria, and decreased abundance of Proteobacteria and Tenericutes in *P. pentosaceus* PR-1 supplementation groups. Besides, HFD detrimentally affected intestinal health, through inducing an increased in the relative abundance of *Ralstonia* and *Aeromonas* in the current study. *Ralstonia* have been shown to cause infections, even more serious, such as osteomyelitis and meningitis (60). *Aeromonas* are widely distributed in aquatic environment and becoming important pathogens which is associated with enteric and extraintestinal infections, such as skin and soft-tissue infections, and lower respiratory tract/urinary tract infections (50). *P. pentosaceus* PR-1 could improve gut health by decreasing the relative abundance of *Ralstonia* and *Aeromonas*. Moreover, *P. pentosaceus* PR-1 supplementation restored abundance of *Cetobacterium* and *Plesiomonas*, which were decreased in HFD-fed zebrafish. Increased proportions of Fusobacteria/*Cetobacterium* were proved to maintain intestinal health of zebrafish through elevating the efficiency of dietary carbohydrate utilization and regulating of glucose metabolism (61, 62). *Cetobacterium* is dominant in the intestines of freshwater and marine fish, and could be applied as potential probiotics in aquaculture (63), due to its ability to improve gut and liver health through decreasing gut inflammation, lipid deposition in liver and serum levels of aspartate aminotransferase and alanine aminotransferase (63), to improve glucose homeostasis through increasing insulin expression (61). Metabolites of *Cetobacterium* such as short-chain fatty acids including acetate, propionate and butyrate and vitamin B12 are dominant in fish intestines and beneficial to fish health (64). The results suggested that *P. pentosaceus* PR-1 improved intestinal health of HFD-fed zebrafish through modulating gut microbiota. In addition, the functional potential of microbiota was also investigated by PICRUSt2 software. According to the results, probiotic administration would inhibit sitosterol degradation. A few studies have found the anti-obesity effect of sitosterol. Sitosterol could lower cholesterol levels and alleviate HFD-induced non-alcoholic fatty liver disease through ameliorating levels of hepatic total lipids, triacylglycerols, cholesterol and liver histopathology, decreasing levels of intestinal bile acids, and increasing the expression of genes involved in lipid metabolism, including HMGCoAR, ABCG5, peroxisome proliferator-activated receptor-α (PPAR-α), and decreasing the expression of CD36 (65). In addition, sitosterol could also reduce inflammatory stress leading to decreased levels of TNF-α, IL-1β, IL-6, and modulate microbiota structure in sheep (66). Therefore, the beneficial effects of *P. pentosaceus* PR-1 in zebrafish may stem from the microbiota metabolites, such as sitosterol. Metabonomics profile would be determined in future to investigate the effects of microbiota metabolites induced by *P. pentosaceus* PR-1 administration, and to further clarify the mechanisms of action of *P. pentosaceus* PR-1 through combination analysis of microbial-omics and metabonomics.

*In vivo* studies reported that HFD would lead to inflammation mediated by IL-1β activation *via* Toll-like receptor 4/NF-κB pathway, and increase expressions of MCP-1, TNF-α, IL-1β, IL-6, MyD88, and NF-κB (2, 36, 48, 67). Consistently, in this study, HFD significantly upregulated hepatic levels and gene expressions of pro-inflammatory cytokines TNF-α, IL-1β, and IL-6, suggesting that HFD induced intestinal inflammation. The hepatic levels and expressions of IL-1β and IL-6 were significantly reduced with a probiotic supplementation, indicating the suppressing inflammation role of *P. pentosaceus* PR-1. The immunoregulatory effect of *P. pentosaceus* was also proved in a murine study that *P. pentosaceus* PP04 relieved the HFD-caused gut inflammation by inhibiting NF-κB/Nrf2 pathway and expressions of TNF-α, IL-1β, IL-6 (36). In the current study, *P. pentosaceus* PR-1 may exert anti-inflammatory effects through restoring the abundance of *Cetobacterium*. Previous studies have found *Cetobacterium* could decrease gut and liver inflammation though stimulating production of butyrate which could inhibit NF-κB, and subsequently lead to decreased expression of pro-inflammatory cytokine genes, including TNFα, IL-1β (63, 68). In addition, *P. pentosaceus* PR-1 may reduce intestinal inflammation through inhibiting the degradation of sitosterol (66). Subsequently, western blot would be performed to determine the alterations in inflammation pathways, including Toll-like receptor 4/NF-κB and NF-κB/Nrf2, induced by *P. pentosaceus* PR-1.

Moreover, previous studies showed HFD could detrimentally affect gut health by inducing intestinal injury (48, 56). Intestinal TJ proteins, including occludin, claudin, and ZO-1 play an important role in maintaining gut permeability and barrier function (48). Previous studies reported that as the TJ expression levels decreased induced by HFD, the gut permeability increased, which lead to increased level of serum endotoxemia and impaired immune response (42, 48). We investigated whether probiotic *P. pentosaceus* PR-1 could improve gut barrier function in HFD-fed zebrafish. The results suggested that *P. pentosaceus* PR-1 supplementation restored the expressions of occludin, claudin, and ZO-1, indicating that *P. pentosaceus* PR-1 exerted benefits to gut integrity and gut health which were impaired by HFD. This was similar to an *in vivo* study, which reported that the expressions of TJ were significantly up-regulated in obesity C57BL/6N mice model induced by HFD (36). The protective effects of *P. pentosaceus* PR-1 on gut barrier function may be associated with its stimulation effect on activity of *Cetobacterium*, which have been found to up-regulate expression of claudin occluding and tight junction protein 2a, playing important role in tight junction assembling and integrity maintaining (68). In addition, *Cetobacterium* could up-regulate the expression of hypoxia-inducible factor-1α, which protects gut barrier function through inhibiting oxidative stress, down-regulating intestinal inflammation, and maintaining intestinal microbiota homeostasis (69, 70). To date, several signaling pathways have been found to be involved in regulation of intestinal barrier function, including the protein kinase C (PKC), PKA, PKG, protein phosphatases PP1, PP2A, PP2B, Rho, myosin light chain kinase, mitogen-activated protein kinase, phosphatidylinositol 3-kinase/Akt, and Wnt/β-catenin pathways (71, 72). Subsequently, western blot and immunofluorescence analysis would be performed to investigate the effects and mechanisms of *P. pentosaceus* PR-1 on levels of intestinal TJ proteins.

SREBP1 is a transcription factor that stimulates hepatic fatty acid biosynthesis *via* upregulation of lipogenesisrelated genes, such as FAS (35). The uncontrolled activation of SREBP-1c is associated with hepatosteatosis, insulin resistance and pro-inflammatory signaling cascade (73). Circulating leptin levels are related to body fat mass. The binding of leptin to its hypothalamic receptor regulates food intake and caused the suppression of fat accumulation and reduction in body weight (57). However, HFD-induce obesity is associated with leptin resistance, which is characterized by high circulating leptin levels (57). Our study found HFD-fed zebrafish significantly up-regulated expression of SREBP1, FAS, and LEPTIN, with the strongest up-regulatory effect on Leptin. *P. pentosaceus* PR-1 supplementation exerted anti-adipogenic activity in HFD-fed zebrafish through regulating adipogenesis-related genes. A murine study also reported a similar finding with *P. pentosaceus* PP04 supplementation to an HFD-induced hyperlipidemia mice model (36). Moreover, we observed that HFD induced liver steatosis and injury in zebrafish, which was obviously reversed by *P. pentosaceus* PR-1 supplementation. The liver plays critical role in nutrients metabolism including fat, and fatty acids which would be stored at triglyceride when exceed the removal capacity of liver (74). Therefore, HFD would lead to cholesterol accumulation in liver, and levels of hepatic cholesterol could be used to indicate the cholesterol absorption. The current study observed the hepatic levels of triglyceride and cholesterol was elevated by HFD and down-regulated by probiotic supplementation, indicating that probiotic could attenuate lipid accumulation and abnormalities in liver. A higher level of LDL is associated with the incidence and severity of diabetes, non-alcoholic fatty liver disease, cardiovascular disease (75). Down-regulatory effect of probiotic administration on LDL was observed in this study, suggesting the beneficial effect of probiotic on lipid homeostasis of liver. The liver protective and anti-obesity effect of *P. pentosaceus* PR-1 may result from the increased abundance of *Cetobacterium* and its metabolites such as short chain fatty acids, which could inhibit the formation of lipid droplets and the expression of lipogenesis genes, and stimulate the expression of lipolysis genes (63, 76). Besides, several studies have explored that probiotics could regulate lipid metabolism through several signaling pathways including PPAR-α, PPAR-γ, carnitine palmitoyltransferase 1, lipoprotein lipase, and CCAAT enhancer-binding protein-α, which are involved in regulation of oxidative respiration, fatty acid β-oxidation, adipocyte proliferation, and differentiation (77, 78). Subsequently, to further clarify the liver protective and anti-obesity effect of *P. pentosaceus* PR-1, signaling pathway expression levels involved in lipid metabolism would be investigated in the future.

## 5. Conclusion

This study is the first to report that *P. pentosaceus* PR-1 improves intestinal health in zebrafish. *P. pentosaceus* PR-1 regulated the gut microbiota, suppressed intestinal inflammation, improved gut barrier function, inhibited adipogenesis. Since zebrafish has become a well-established vertebrate model, our findings suggest the application of *P. pentosaceus* PR-1 for improving lipid metabolic disorder. Although the beneficial effects of *P. pentosaceus* PR-1 on intestinal health of zebrafish were observed, the current study did not proof the underlying mechanisms. Therefore, it would be of significant importance to further explore the mechanisms of action of *P. pentosaceus* PR-1 in depth.

## Data availability statement

The original contributions presented in the study are publicly available. This data can be found at: https://www.ncbi.nlm.nih.gov/bioproject/PRJNA899980.

## Ethics statement

The animal study was reviewed and approved by Experimental Animal Welfare and Ethics Committee of the Shanxi University.

## Author contributions

YL designed the study. DZ and JL performed most of the experiments. XS and FG performed statistical analysis. YL wrote the manuscript. HD and LD conducted microbiota analysis. YL and XW contributed to data organization or analysis. YL and CW supervised the study. All authors contributed to comments and revision on the manuscript and approved the final manuscript.

## Supplementary material

Supplementary Figure 1 Manhattan map of differentially abundant amplicon sequence variants (ASVs) based on metagenomeSeq analysis. The plot is ordered by taxonomy line and the colors correspond to different phyla. The y-axis displays the negative log of the p-value, and higher values indicate increased statistical significance. Each dot or circle in the coordinate system represents one ASVs, and the size indicates its relative abundance. The dotted line separates the significant difference (above) from the insignificant ASVs. Significantly different points are marked with colors, and insignificant ones are gray circles. Significantly upward alterations are displayed with colored solid dots; the color of the dots suggests phylum name of x-axis. **(A)** H group compared to C group. **(B)** HP group compared to H group.

Supplementary Figure 2 LEfSe analysis explored the discriminative microbiota between C and H groups **(A)**, and discriminative microbiota between H and HP groups **(B)**, *p* < 0.05 was used as a threshold for LEfSe analysis.

Supplementary Table 1 Anosim (comparing C and H group).

Supplementary Table 2 Anosim (comparing H and HP group).
