# Supplementary figures and images for: Pediococcus pentosaceus PR-1 modulates high-fat-died-induced alterations in gut microbiota, inflammation, and lipid metabolism in zebrafish
Source: Front Nutr. 2023 Feb 1;10:1087703. doi: 10.3389/fnut.2023.1087703 (PMC9929557; doi:10.3389/fnut.2023.1087703)

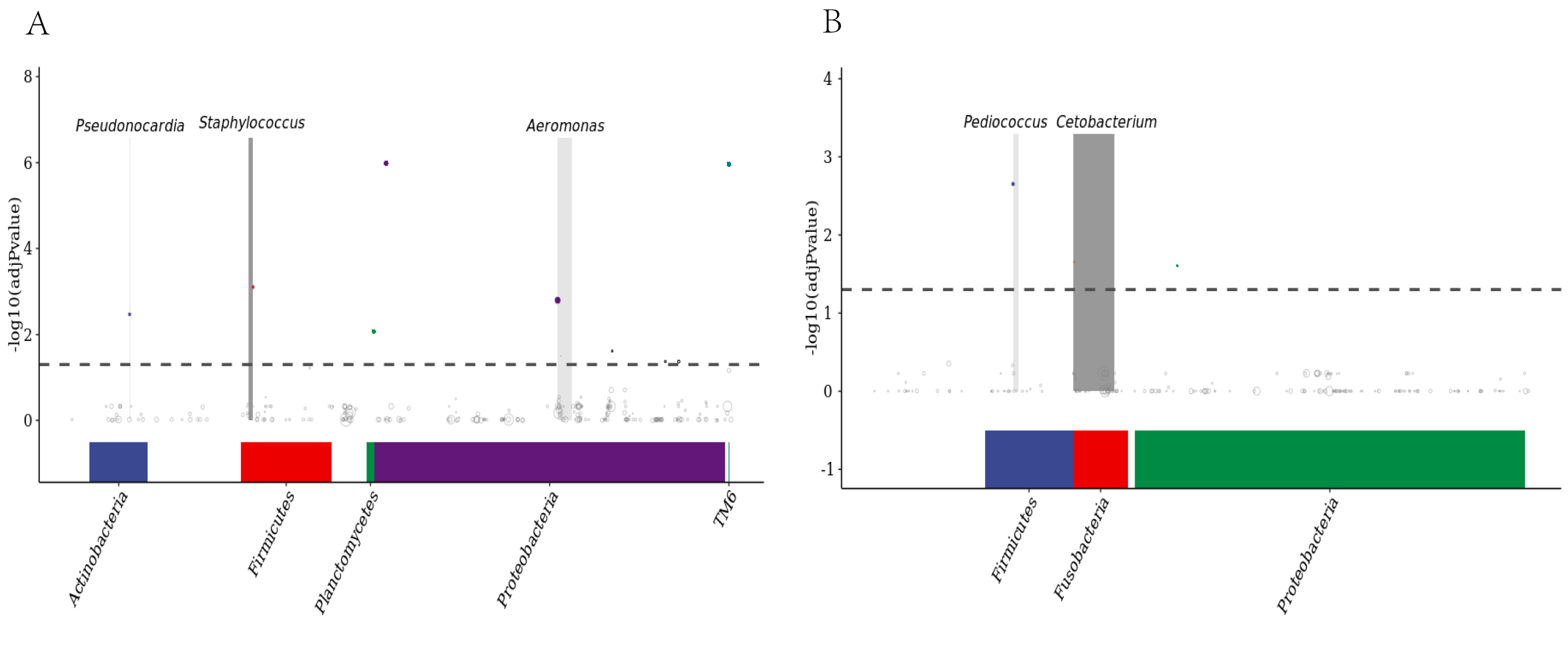

Supplement: Supplementary Figure 1 — Manhattan map of differentially abundant amplicon sequence variants (ASVs) based on metagenomeSeq analysis. The plot is ordered by taxonomy line and the colors correspond to different phyla. The y-axis displays the negative log of the p-value, and higher values indicate increased statistical significance. Each dot or circle in the coordinate system represents one ASVs, and the size indicates its relative abundance. The dotted line separates the significant difference (above) from the insignificant ASVs. Significantly different points are marked with colors, and insignificant ones are gray circles. Significantly upward alterations are displayed with colored solid dots; the color of the dots suggests phylum name of x-axis. (A) H group compared to C group. (B) HP group compared to H group. [file Image_1.tif]

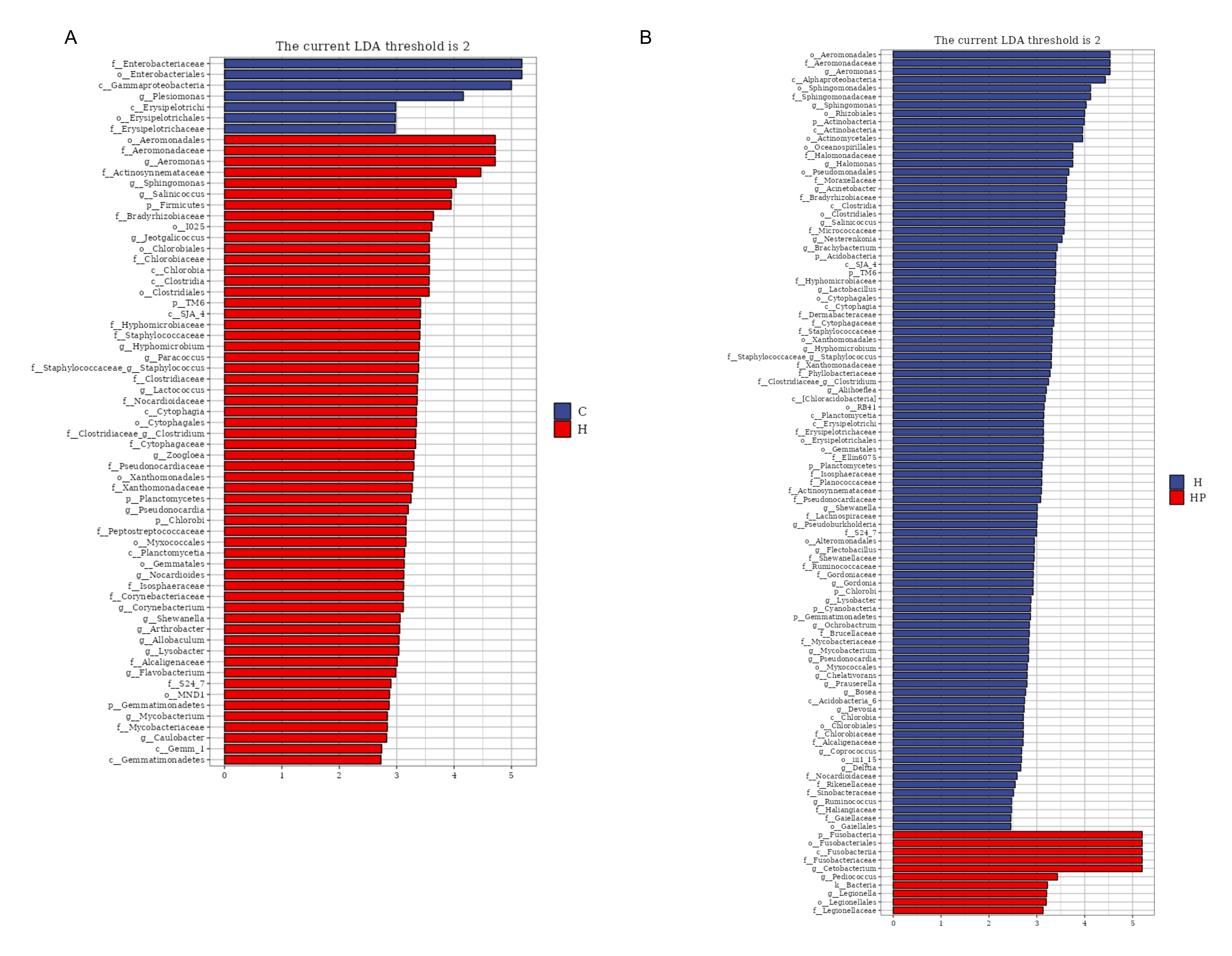

Supplement: Supplementary Figure 2 — LEfSe analysis explored the discriminative microbiota between C and H groups (A), and discriminative microbiota between H and HP groups (B), p < 0.05 was used as a threshold for LEfSe analysis. [file Image_2.tif]
