# Supplementary material for: Pediococcus pentosaceus PR-1 modulates high-fat-died-induced alterations in gut microbiota, inflammation, and lipid metabolism in zebrafish
Source: Front Nutr. 2023 Feb 1;10:1087703. doi: 10.3389/fnut.2023.1087703 (PMC9929557; doi:10.3389/fnut.2023.1087703)
Supplement: Supplementary Table 1 — Anosim (comparing C and H group). [file Data_Sheet_1.docx]

Table S 1Anosim (Comparing C and H group).

| Method name | R | p-value | Permutations |
| --- | --- | --- | --- |
| Anosim | 0.181202 | 0.065 | 999 |

Table S 2 Anosim (Comparing H and HP group).

| Method name | R | p-value | Permutations |
| --- | --- | --- | --- |
| Anosim | 0.295933 | 0.016 | 999 |
